# 24th Collegium Ramazzini Statement: Prevention of Work-Related Infection in the COVID-19 Pandemic

**DOI:** 10.5334/aogh.2929

**Published:** 2020-07-07

**Authors:** The Fellows of the Collegium Ramazzini

**Affiliations:** 1 The Fellows of the Collegium Ramazzini, IT

## Abstract

**Background:**

Workers whose occupations put them in contact with infected persons and the public are at increased risk of COVID-19 infection.

**Recommendations:**

The Collegium Ramazzini calls on governments at all levels to protect worker health by strengthening public health systems; maintaining comprehensive social insurance systems; establishing policies that presume all COVID-19 infections in high-risk workers are work-related; enforcing all occupational health standards; and developing pandemic preparedness plans. The Collegium Ramazzini calls on all employers – large and small, public and private – to protect the health of all workers by developing disease preparedness plans; implementing basic infection control measures; establishing disease identification and isolation policies; reducing hazardous exposures; supporting personal protective equipment (PPE) programs; and restricting unnecessary travel.

**Conclusion:**

Governments and employers have legal obligations to protect worker health. They are not relieved of these duties during pandemics.

The Collegium Ramazzini is an independent, international society comprised of 180 physicians and scientists from 35 countries. Its mission is to increase scientific knowledge of the environmental and occupational causes of disease and to transmit this knowledge to decision makers, the media, and the global public to prevent disease, promote health, and save lives.

**The Collegium Ramazzini calls urgently for preventive measures internationally and in every country to reduce risk of COVID-19 infection in workers.**

The COVID-19 pandemic has affected every country in the world, caused confirmed illness in more than 3 million people, unconfirmed disease in millions more, and more than 200,000 deaths. At present, there is no vaccine and no medical treatment for COVID-19. Therefore, pandemic control must rely entirely on measures that reduce the spread of infection, flatten the epidemic curve, and gain time to develop more effective responses.

Workers whose occupations put them in contact with infected persons and the public are at greatly increased risk of disease and death and have suffered disproportionately in the COVID-19 pandemic. Workplaces have repeatedly been the source of serious outbreaks.

Protection of all workers, and especially workers who continue to provide essential services during the pandemic, as well as vulnerable workers, must be a top priority at every stage.

## High-Risk Workers

Workers whose occupations put them in contact with infected persons and the public are at greatly increased risk of COVID-19 infection. These workers require heightened protection. A partial listing of high-risk workers follows:

- **Very High Risk:** Health care workers; paramedics; police; firefighters; airline personnel; transport workers; drivers; sales and service personnel; cleaners; mortuary workers; migrant workers; volunteers; and religious professionals.
- **High Risk:** Security service workers; hotel and food service workers; cruise industry workers; military personnel pressed into pandemic service; workers in infrastructure, manufacturing, meatpacking, construction, mining, and other occupations with crammed workplaces and poor provision of occupational and personal hygiene measures.
- **Workers at Increased Vulnerability:** Older workers; workers with underlying medical conditions, such as hypertension, obesity, heart disease and cancer; workers occupationally exposed to dusts, gases and fumes; workers of low socio-economic status; workers exposed to high levels of ambient air pollution; and workers in developing countries.

**The Collegium Ramazzini calls on governments at all levels—national, state or provincial, and local—and on all employers—large and small, public and private—to fulfill their responsibilities to protect the health of all workers in the COVID-19 pandemic.**

## Responsibilities of Governments

### Maintain and Strengthen Public Health Systems

Governments have responsibility to protect workers’ health and the health of populations by (1) maintaining disease surveillance systems that track the spread of disease and obtain information on the industry and occupation of each sick and injured worker; (2) supporting epidemic intelligence services and laboratories that warn of impending pandemics; (3) organizing and leading responses to prevent disease and death; and (4) communicating accurate, evidence-based information to the public that openly acknowledges limits and uncertainties in current knowledge.

### Maintain and Strengthen Comprehensive Social Insurance Systems

Governments have a responsibility to protect the health of workers by sustaining comprehensive social insurance systems that include health insurance, unemployment insurance, contributions to the pension system, and provision of wages and benefits during illness and isolation. Workers must be permitted to report sick and enter quarantine without fear of losing wages or benefits. All workers must be covered by such systems, including those in precarious working conditions such as migrant workers, volunteers, and the self-employed.

### Enforce Regulations

Governments have a responsibility to regularly inspect workplaces and to ensure that all employers fulfill their legal and moral duty to care for their workers. Governments must ensure that employers protect their workers against occupationally acquired infection as well as against occupational and environmental exposures that increase risk of infection and severity of disease. Governments are not relieved of these responsibilities during pandemics.

### Establish Presumption Policies

Governments can further protect the health of workers and the population by officially defining COVID-19 infection as an occupational disease and establishing the presumption that any COVID-19 infection in a worker in a high-risk occupation or industry is work-related.

### Pandemic Preparation

Governments must prepare for future pandemics by investing in public health and occupational health programs, maintaining disease surveillance systems, and maintaining adequate stockpiles of emergency medicines and critical supplies.

### Post-Pandemic Planning

As numbers of new cases decline in the late stages of a pandemic, governments must develop protocols for systematically relaxing infection control procedures and reopening schools and businesses, beginning with lower-risk activities. These protocols must monitor numbers of new cases and the availability of medical resources, continuously calibrate the pace of restart until a vaccine or an effective treatment has become widely available, and be prepared to pause or reverse reopening if a new wave of infections emerges. During this phase, governments must continue to sustain comprehensive social insurance systems that protect workers from unemployment, job instability, and stress caused by the pandemic.

## Responsibilities of Employers

Employers have a legal and moral duty to protect their workers against occupationally acquired COVID-19 infection. Employers are not relieved of these responsibilities during pandemics. Specific duties of employers in all at-risk industries are the following.

### Develop an Infectious Disease Preparedness and Response Plan

This is a key component of pandemic planning and preparation. It includes designation of an infection control officer, provision of training to all at-risk workers, eliminating adverse working conditions that predispose to spread of infection, such as crowding and extreme work hours, and development of pandemic contingency plans for staggered work shifts, provision of meals, and teleworking. Pandemic preparation by employers parallels pandemic planning by governments, and the two must link.

### Implement Basic Infection Control Measures

These include regularly disinfecting common areas and shared equipment and tools, sneezing and coughing etiquette, frequent handwashing, and physical separation during work hours and in breaks.

### Establish Disease Identification and Isolation Policies

Develop policies and procedures for prompt identification, testing, and isolation of workers with known or suspected infection.

### Reduce hazardous occupational exposures

Reduce occupational exposures to dusts, gases, and fumes that increase risk and severity of infection.

### Provide Appropriate Exposure Controls

Engineering and administrative controls must be given highest priority. PPE and behavioral controls are used only when and if there is no other feasible option.

### Support Effective Personal Protective Equipment (PPE) Programs

Effective PPE programs not only provide equipment, but also include training, selection, fit testing, proper use, disposal, and disinfection of all PPE items.

### Restrict Unnecessary Travel

Establish travel policies that allow only necessary travel and require isolation of all travelers for 14 days upon return from any domestic or international air travel. Exemptions may be granted for travelers documented through validated, reliable testing to have immunity against COVID-19.

### Communication

Communicate accurate, evidence-based information to workers openly and frequently through a single spokesperson, ideally the infection control officer.

### Exceptions for Immunity

Exceptions may be made for workers who are found to have immunity against COVID-19.

**Further elaboration is provided in the attached Technical Appendix and its Annexes.**

The COVID-19 pandemic has demonstrated a woeful lack of preparedness in many governments, health care organizations, and employers. This is the consequence of decades of neglect of public health and occupational health. Staffs of health agencies have been cut and budgets slashed. Emergency stockpiles of medications and supplies have been disbanded. Training has been neglected. Contingency plans have not been developed. Leadership capacity has eroded. **We are all paying the price of this neglect. Workers are paying the highest price.**

## Additional File

The additional file for this article can be found as follows:

- **Technical Appendix.** http://www.collegiumramazzini.org

## Co-publishing Statement

Several other journals around the world are co-publishing this Statement as there is an urgent need to advise the health professions and the global public of this critical information.

